# MiRNA Regulation of MIF in SLE and Attenuation of Murine Lupus Nephritis With miR-654

**DOI:** 10.3389/fimmu.2019.02229

**Published:** 2019-09-19

**Authors:** Yang Tu, Ruru Guo, Jia Li, Suli Wang, Lin Leng, Jun Deng, Richard Bucala, Liangjing Lu

**Affiliations:** ^1^Department of Rheumatology, School of Medicine, Renji Hospital, Shanghai Jiao Tong University, Shanghai, China; ^2^Section of Rheumatology, Allergy and Immunology, Yale University School of Medicine, New Haven, CT, United States; ^3^China-Australia Centre for Personalised Immunology, Renji Hospital, Shanghai Jiao Tong University School of Medicine, Shanghai, China; ^4^Department of Rheumatology, Shanghai Institute of Rheumatology, Renji Hospital, Shanghai Jiao Tong University School of Medicine, Shanghai, China

**Keywords:** miRNA, MIF, SLE, pristine, lupus nephritis

## Abstract

**Objective:** Macrophage Migration Inhibitory Factor (MIF) is involved in the pathogenesis of systemic lupus erythematosus (SLE) and lupus nephritis (LN). MicroRNAs (miRNAs) play important roles in LN but whether specific miRNAs regulate the expression of MIF in LN is unknown. We explore specific miRNAs that can regulate MIF expression, and investigate miR-654 for the treatment of experimentally-induced murine lupus nephritis.

**Methods:** Sera samples from 24 SLE patients and 24 controls were collected to measure the MIF concentration and its correlation with disease activity. A luciferase reporter assay was used to explore the target of miR-654. ELISA was used to detect the downstream cytokines regulated by miR-654 and MIF. Western blot was applied to measure the impact of miR-654 inhibition on downstream MIF signaling. The therapeutic efficacy of miR-654 was tested in the pristine-induced lupus mouse model. We further measured miR-654 expression and analyzed its relationship with MIF expression in SLE patients.

**Results:** The serum MIF level was increased in SLE patients (*p* < 0.001) and positively correlated with the SLEDAI score (*r* = 0.5473; *p* = 0.0056). MiR-654 inhibited MIF and downstream inflammatory cytokine production by selectively inhibiting the phosphorylation of ERK and AKT. Activation of miR-654 reduced IL-1β, IL-6, IL-8, and TNF-α production, reduced gomerulonephritis, and decreased MIF, IgG, and C3 expression in murine lupus glomeruli. Furthermore, MIF was negatively correlated with miR-654 expression (*r* = −0.4644; *p* = 0.0222) in SLE patients.

**Conclusion:** MiR-654 negatively correlated with MIF and disease activity in patients with SLE. MiR-654 inhibits MIF expression via binding to MIF 3'UTR, selectively suppresses the phosphorylation of ERK and AKT, and reduces downstream inflammatory cytokine production. *In vivo* miR-654 treatment decreases MIF and downstream cytokine production and ameliorates murine lupus nephritis.

## Introduction

Systemic lupus erythematosus (SLE) is a prototypic immune complex disease that affects multiple organ systems. The immunologic features of SLE include autoantibodies directed against nuclear components and elevated pro-inflammatory cytokines, such as type I interferon (IFN), IL-1β, IL-6, IL-8, TNF-α, and BAFF (1). Clinical interventions targeting multiple mediators such as IFNα, IL-1β, TNF-α, IL-6, or BAFF have been largely disappointing (2), suggesting that other effector pathways may play a more dominant role in SLE pathogenesis or disease manifestations (3, 4).

Macrophage migration inhibitory factor (MIF) and its receptors are expressed in elevated levels by different cell types in SLE (5), and circulating MIF level correlates with disease activity (6). High expression *MIF* alleles also are linked to disease severity in different populations (7, 8). MIF binds to a receptor complex comprising CD74 and CD44 to activate the phosphorylation of ERK and AKT, leading to the expression of pro-inflammatory cytokines such as IL-1β, IL-6, IL-8, TNF-α, and others (9–11). Glomerular injury and renal disease manifestations are reduced in MRL/*lpr Mif*^−/−^ mice (12), and pharmacologic MIF antagonists protect against glomerulonephritis in lupus-prone MRL/*lpr* or NZB/NZW F1 mice (13). Our group previously reported that MIF mediates steroid resistance in SLE by upregulating the expression of IκB, the cytosolic binding partner for NFκB (14). The precise mechanisms underlying MIF expression and pathologic function nevertheless remain poorly understood in SLE.

The microRNAs (miRNAs) are a class of small (21–25 nucleotides) non-coding RNAs that regulate gene expression by promoting mRNA degradation or by translational repression at the 3'UTR of target genes (15). Extensive studies demonstrate that some miRNAs are pathogenic, and others are protective in SLE patients (16–18). Despite these data, supporting the role of MIF-dependent pathways in LN, the exact mechanism(s) by which miRNAs modulate LN and regulate the expression of MIF remain unknown.

In this study, We identify miR-654 as a negative regulator of MIF expression that reduces MIF-dependent AKT and ERK phosphorylation and downstream, pro-inflammatory cytokine expression. These findings suggest that targeting miR-654 may be a useful approach for the improvement of lupus nephritis.

## Materials and Methods

### Human Study Subject Samples

This research was approved by the Research Ethics Board of Renji Hospital, Shanghai Jiao Tong University School of Medicine. Blood samples from SLE patients and healthy controls (HC) were collected with the informed and written consent of all patients and healthy controls. Patients diagnosed with SLE were based on the American College of Rheumatology 1982 revised criteria for SLE (19), and the SLE Disease Activity Index (SLEDAI) was used to access the disease activity of SLE patients (20). Among the 24 patients, 15 patients had lupus nephritis proven by biopsy. Healthy controls were free of autoimmune disease, tumor, infection, or other diseases that influence the expression of inflammation cytokines. Both patients and healthy controls were between the ages of 18 and 65.

### Cell Culture, Transfection, and Stimulation

Peripheral blood mononuclear cells (PBMC) were isolated from whole blood by using Ficoll-Paque gradient centrifugation (Sigma), at 400 g for 20 min at 20°C. The PBMCs then were collected and washed twice with sterile PBS. Jurkat, RAW264.7, and THP-1 cell lines were purchased from the cell bank of Shanghai Institutes for Biological Sciences at the Chinese Academy of Sciences. miRNA mimic and miRNA inhibitor were obtained from GenePharma. The sequence of the mimic, inhibitor, and control can be seen in Supplementary Table 1. Lipofectamine RNAiMAX (Invitrogen) was used to transfect miRNAs into cell lines according to the manufacturer's instructions. The RAW264.7 cells were transfected with miRNA mimic or negative control for 24 h, stimulated with MIF (100 ng/ml) for 6 h, and cytokine expression was analyzed. THP-1 monocytes were incubated with PMA (100 ng/ml) for 24 h to obtain THP-1 derived macrophages. After RAW264.7 and THP-1 derived macrophages were transfected with miRNA mimic or negative control for 24 h, MIF (100 ng/ml) was used to stimulate the macrophages for 15 or 30 min; the cells then were harvested to detect the expression of ERK, pERK, AKT, pAKT, PI3K, Jun, and pJun by western blot.

### Quantitative Real-Time Polymerase Chain Reaction (qPCR)

Total RNA was extracted using TRIzol reagent (Invitrogen) according to the manufacturer's instruction. PrimeScript RT Reagent kit (Takara) and SYBR Premix Ex Taq RT-PCR kit (Takara) were used to detect the quantities of mRNA expression. The fold-change of each gene was normalized to GAPDH as the internal control using 2–ΔΔCt method. TaqMan MicroRNA Assay kit (Applied Biosystems) and specific TaqMan probes (Applied Biosystems) were used to detect the expression of miRNAs. RNU6 was used as the internal control for humans, and sno202 was used as the internal control for mice.

### Western Blot

Cells were harvested in lysis buffer and heated at 95°C for 10 min; the cell lysates then were subjected to 15% sodium dodecyl sulfate-polyacrylamide gel electrophoresis (SDS-PAGE) and transferred to PVDF membranes (Millipore). After blocking with 8% skim milk, the membranes were incubated with primary antibodies directed against phosphorylated ERK (Thr202/Tyr204), ERK, phosphorylated Jun (Ser73), Jun, AKT, PI3K, GAPDH, tubulin (all purchased from Cell Signaling Technology), MIF (R&D, Catalog # 289-MF) at 4° overnight. After addition of HRP-conjugated secondary antibody (1: 2,000, Cell Signaling Technology), the membranes were washed and the protein bands visualized by chemiluminescence detection kit (Thermo) and quantified with density-analysis software. Anti-tubulin antibody was used as a sample loading control.

### Enzyme-Linked Immunosorbent Assay (ELISA)

Protein levels of MIF in sera, MIF, IL-1β, IL-6, IL-8, TNF-α in cell supernatants, and mouse sera MIF, IL-1β, IL-6, IL-8, TNF-α were measured with enzyme-linked immunosorbent assay (ELISA) kit (R&D). Antinuclear antibodies (ANA), anti-dsDNA antibodies and C3 in mouse sera were measured by ELISA kits (Alpha Diagnostic International, San Antonio, TX). Urine protein expression levels were examined by ELISA kit (Sigma-Aldrich). All ELISAs were carried out according to the manufacturer's instruction.

### Luciferase Assays

A psi-check2 vector containing the MIF 3'UTR sequence was constructed. As the MIF 3'UTR sequence is very short, the 3'UTR sequence was synthesized directly. The sense primer sequence is:

CTCGAGGAGCCGCAGGGACCCACGCTGTCTGCGCTGGCTCCACCCGGGAACCCGCCGCACGCTGTGTTCTAGGCCCGCCCACCCCAACCTTCTGGTGGGGAGAAATAAACGGTTTAGAGACT.

and the anti-sense primer sequence is:

GCGGCCGCAGTCTCTAAACCGTTTATTTCTCCCCACCAGAAGGTTGGGGTGGGCGGGCCTAGAACACAGCGTGCGGCGGGTTCCCGGGTGGAGCCAGCGCAGACAGCGTGGGTCCCTGCGGCTC.

The psi-check2 vector was digested with Not I and Xho I for 1 h at 37° overnight and the digested vector was used to assemble the MIF 3'UTR sequence. The psi-check2 MIF 3'UTR-mutant vector was constructed by using the TaKaRa MutanBEST Kit (Takara). For the Luciferase assay, 293T cells were seeded at 2 × 10^5^ cells/well in a 24-well plate 1 day before transfection and then transfected with a mixture of 100 ng psi-check2 vector, psi-check2 MIF 3'UTR vector, or psi-check2 MIF 3'UTR-mutant vector together with 100 nM miR mimic, inhibitor, or negative control using Lipofectamine 3000 (Invitrogen). The cells were harvested 24 h later and the Dual-Luciferase Reporter Assay System (Promega) was used to detect the fluorescence activity. Renilla luciferase was used to normalize the firefly luciferase.

### Mice

Female BALB/c mice were purchased from Shanghai SLAC Laboratory Animal Co., Ltd. (Shanghai, China) and were maintained in the animal facility with *ad libitum* feeding at the Shanghai Renji Hospital. Female mice at 8–10 weeks were used for experiments. The lupus mouse model was induced by intraperitoneal administration of 0.5 ml pristine (Sigma) one time (21). Each group was random allocated six mice. After 1 month induction with pristine, mice were treated with miR-654 mimic or miR-654 inhibitor three times every 4 weeks. MiRNA mimic and miRNA inhibitor were incubated with Lipofectamine™ RNAiMAX(Thermo Fisher, Catalog Number: 13778150) in room temperature for 5 min, then 200 μl mixture was injected in tail vein. After 4 months induction with pristine, 24 h urines were collected three times every 4 weeks. Mice were sacrificed, tissues and blood were harvested in the sixth month after induced by pristine. All animal experiments complied with the relevant institutional guidelines of animal ethics from Renji Hospital, Shanghai Jiao Tong University, School of Medicine.

### Kidney Histology

Kidney tissues were fixed in 4% paraformaldehyde, embedded in paraffin, cut into 4 μm thick sections and stained with periodic acid–Schiff reagent. Assessments of renal lesions by the activity score described for humans (including glomerular proliferation, mesangial matrix expansion, fibrinoid necrosis, crescent formation, infiltrating leukocytes, hyaline deposits), which graded from 0 to 3 (0, no lesions, 1, lesions in <30% of glomeruli, 2, lesions in 30–60% of glomeruli, and 3, lesions in >60% of glomeruli). Tissues were scored by two kidney pathologists in a blinded manner.

### Statistical Analysis

Categorical data were expressed by counts or percentages, and compared between different groups using the chi-squared test or continuity correction as appropriate. The continuous variables were presented as the mean (standard deviation), and were analyzed by Student's *t*-test or the Mann-Whitney *U*-test as appropriate. All the correlation analyses were performed by Spearman correlation. A *p* < 0.05 was considered statistically significant. All analyses were performed using Graph Pad Prism Version 6.

## Results

### MIF Is Highly Expressed in SLE Patients and Positively Related to Disease Activity Score

The clinic characteristics of SLE patients and healthy were listed in Table 1. The average age of SLE patients was 40.08 ± 2.83, and the average age of Ctrl was 39.75 ± 2.68, and there were 22 women and 2 men both in the SLE patients and Ctrl group. The duration of disease was 4.65 ± 1.04 years, the SLEDAI score was 12.75 ± 1.23, and the 24 h urinary protein in SLE patients was 2.614 ± 0.803 g.

**Table 1:** Characteristics of SLE patients and healthy controls.

**Index**	**SLE**	**Healthy control**
Sex (Female:Male)	22:2	22:2
Age (year)	40.08 ± 2.828	40.25 ± 2.755
SLEDAI	12.75 ± 1.231	
Disease course(year)	4.65 ± 1.038	
24h Urine protein(g)	2.614 ± 0.803	
C3 (g/L)	0.486 ± 0.065	
C4 (g/L)	0.169 ± 0.044	
Anti-dsDNA	140.3 ± 46.68	
Anti-nuclear Antibody positive	24 (100%)	
ESR	48.67 ± 6.043	
CRP	5.934 ± 1.681	
GC (mg/d)	75 ± 19.98	
**Clinical presentation**		
Malar Rash	13 (54%)	
Oral Ulcers	10 (41.7%)	
Hair loss	17 (70.8 %)	
Photosensitivity	9 (37.5%)	
Arthritis	18 (75 %)	
Hematological Disorder	12 (50%)	
Neurological Disorder	2 (8.3%)	
**Immunomodulating drugs**		
CTX	4 (16.7%)	
MMF	11 (45.8%)	
FK506	3 (12.5%)	
HCQ	24 (100%)	
MTX	15 (62.5%)	

*The continuous variables were presented as the mean ± standard deviation*.

**Figure 1:**
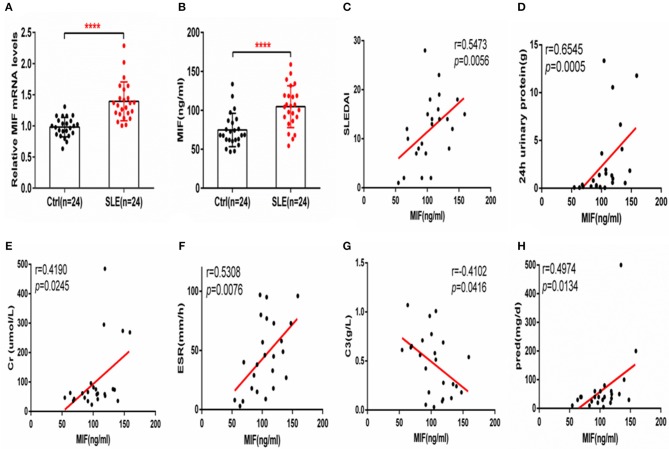
MIF expression is increased in PBMCs and sera from SLE patients and correlates positively with SLEDAI score. **(A)** Quantitative polymerase chain reaction (qPCR) analysis of MIF expression in PBMCs from SLE patients (*n* = 24) compared with healthy controls (*n* = 24). **(B)** Circulating serum MIF levels in SLE patients (*n* = 24) and healthy controls (*n* = 24). **(C)** Serum MIF levels from SLE patients correlate positively with SLEDAI score. **(D)** Serum MIF levels from SLE patients correlate positively with 24 h urinary protein (*n* = 24). **(E)** Serum MIF levels from SLE patients correlate positively with serum creatinine (*n* = 24). **(F)** Serum MIF levels from SLE patients correlate positively with ESR (*n* = 24). **(G)** Serum MIF levels from SLE patients correlate negatively with serum C3 (*n* = 24). **(H)** Serum MIF levels from SLE patients correlate positively with the dosage of steroids. **(A,B)** Were analyzed by Mann-Whitney test. **(C,D)** Were analyzed by non-parametric Spearman's correlation. ****P ≤ 0.0001 vs. negative control.

Macrophage migration inhibitory factor (MIF) mRNA was more highly expressed in PBMCs from SLE patients than from healthy controls (*P* < 0.001, Figure 1A), and serum MIF expression was higher in SLE group (*P* < 0.001, Figure 1B). A positive correlation between serum MIF expression and SLEDAI score in SLE patients (*P* < 0.001) was observed, in agreement with prior reports (6, 7) (Figure 1C). Both 24 h urinary protein and serum creatinine were positively correlated to the serum MIF expression (*P* < 0.05, Figures 1D,E). We also found that ESR was positively related with MIF expression (*P* < 0.01, Figure 1F). However, MIF expression was negatively related with C3 expression, an index of disease activity (*P* < 0.05, Figure 1G). It is well-known that steroids affect MIF levels, so we analyzed the dosage of steroids at the time of blood collection in all patients, and found that MIF expression is positively related with the dosage of steroids (*P* < 0.05) (Figure 1H). We also analyzed the analyzed the relationship between five immunosuppression drug (MMF, HCQ, FK506, and MTX) and MIF expression, and found that there was a positively relationship between MMF and MIF expression (*r* = 0.8107, *p* = 0.0127), while there was a negatively relationship between MTX and MIF expression (*r* = −0.1741, *p* = 0.0322; Supplementary Figure 1).

### MIF Expression Is Regulated by miR-654

**Figure 2:**
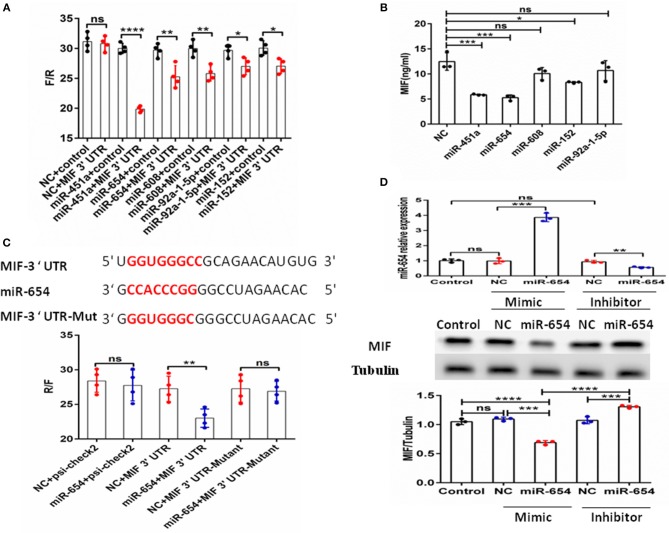
Identification of MIF as a functional target of microRNA-654 (miR-654). **(A)** 293T cells were transfected with the control construct (psiCHECK-2) or with a construct encoding the *MIF* 3′-UTR, in addition to negative control (NC) mimic or miRNA mimic. Luciferase activity in 293T cell lysates was detected 24 h later. **(B)** MIF protein levels after overexpression miR-451a, miR-654, miR-152, or NC in Jurkat T cells as determined by ELISA. **(C)** Predicted binding sites for miR-654, the *MIF* 3′-untranslated region (3′-UTR), and mutant construct. 293T cells were transfected simultaneously with the control vector (psiCHECK-2), a vector containing wild-type *MIF* 3'UTR, mutated *MIF* 3'UTR (mutant), negative control (NC) mimic, or miR-654 mimic. Luciferase activity in 293T cell lysates was detected after 24 h. **(D)** MIF protein levels were reduced by miR-654 mimic and increased by miR-654 inhibitor in human Jurkat T cells, as determined by Western blot. The relative expression of MIF normalized to tubulin in miR-654 group and NC group. **(A–D)** Were analyzed by Mann-Whitney test. *P < 0.05; **P < 0.01; ***P < 0.001; ****P ≤ 0.001 vs. negative control. ns, no significant.

The above data clearly show MIF is connected to SLE disease activity. MiRNAs have been shown to be involved in the pathogenesis of SLE (22). Therefore, we assume that miRNAs may regulate MIF expression. We screened miRNAs that could potentially target the *MIF* 3' UTR as predicted by sequence-based bioinformatics analysis. The tools included miRBase, miRecords, PMRD, miRWalk, TarBase, TargetScan, microRNA. org, miRanda, and RNAhybrid. miR-654 was among the 10 miRNAs predicted to target the *MIF* 3' UTR. The binding sites can be seen in Supplementary Table 2. We constructed a psi-check2 vector containing the 3' UTR of *MIF* (psi-chek2-MIF 3'UTR). The psi-chek2-*MIF* 3'UTR vector, different miRNA mimics, and negative control (NC) were transfected into 293T cells, respectively. Using luciferase reporter assay, we screened candidate miRNAs for inhibition of the chemiluminescence in 293T cells transfected with psi-chek2-*MIF* 3'UTR compared with negative control (Figure 2A). Based on the data about MIF protein expression in different human T cell lines (http://www.genecards.org), we measured MIF protein level by ELISA in Jurkat T cells transfected with five candidate miRNA mimics or negative control and found three miRNAs that inhibit the MIF protein expression (Figure 2B). In our previous research, we have reported that miR-152 attenuated the severity of lupus nephritis by downregulation of MIF (23). In this research, we focus on miR-654.

To verify the specific binding sites of miR-654 in the 3'UTR of MIF, we constructed a mutant variant psi-chek2-*MIF* 3'UTR vector (psi-chek2- *MIF* 3'UTR-Mutant) that we transfected into 293T cells by luciferase reporter assay. We found that miR-654 mimics did not affect the chemiluminescence of 293T cells transfected with psi-chek2-MIF 3'UTR-Mutant fragments in which the 7 base target sites were mutated (Figure 2C). We also examined MIF expression by Western blot in Jurkat T cells after transfection with the miR-654 mimic or miR-654 inhibitor. miR-654 expression was highly increased after transfected with miR-654 mimic, and significantly reduced after transfected with miR-654 inhibitor (Figure 2D). Compared with NC, MIF expression was inhibited after transfection with miR-654 and increased after transfected with miR-654 inhibitor (Figure 2D). Taken together, these results indicate that MIF expression is regulated by miR-654.

### miR-654 Regulates MIF-Dependent Cytokines Expression in Macrophages RAW264.7

**Figure 3:**
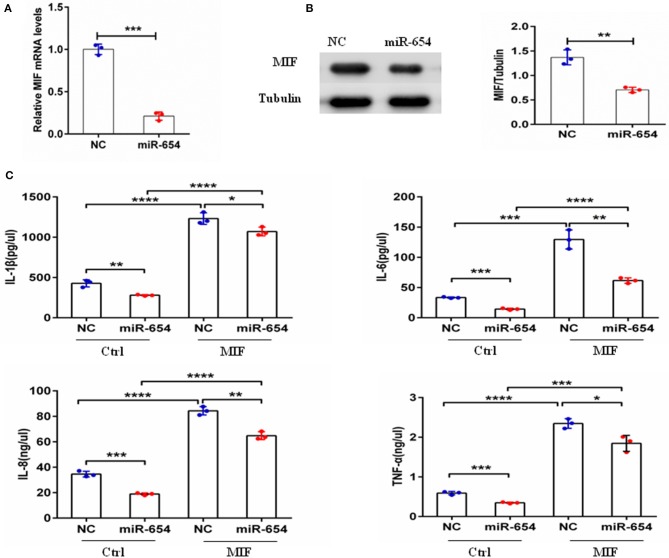
miR-654 regulates MIF-dependent cytokine expression and proliferation of RAW264.7 macrophages. **(A)** qPCR of miR-654 and MIF mRNA showed MIF mRNA downregulation after overexpression miR-654 in macrophages. **(B)** Western blot analysis showing reduced expression of MIF after miR-654 treatment compared to the NC group. The relative expression of MIF is normalized to tubulin. **(C)** The MIF-dependent expression levels of IL-1β, IL-6, IL-8, and TNF-α are significantly reduced after overexpression miR-654 in RAW264.7 macrophages. **(A,B)** Were analyzed by Unpaired student's *t*-test. **(C)** Were analyzed by Mann-Whitney test. *P < 0.05; **P < 0.01; ***P < 0.001; ****P ≤ 0.0001 vs. negative control.

Macrophage migration inhibitory factor (MIF) is an upstream pro-inflammatory mediator that upregulates the expression of IL-1β, IL-6, IL-8, TNF-α, et al (5, 24–27). Referring to prior murine gene-expression profiles [http://biogps.org/#gotowelcome], we analyzed MIF RNA expression in different mouse cell lines and observed highest MIF expression by the transformed RAW264.7 macrophage cell line. To examine if miR-654 can affect MIF downstream inflammatory cytokines expression, we transfected RAW264.7 macrophages with miR-654 mimic or NC 24 h prior to MIF stimulation (duration 6 h). miR-654 expression was significantly increased after transfection with miR-65mimic (Figure 3A). MIF expression was found to be reduced at both the mRNA and the protein levels in response to miR-654 (Figures 3A,B). The expression levels of IL-1β, IL-6, IL-8, and TNF-α also were significantly reduced by over-expression miR-654 compared with NC (Figure 3C).

### MiR-654 Downregulates Inflammatory Cytokines Expression by Inhibiting Phosphorylation of ERK and AKT

**Figure 4:**
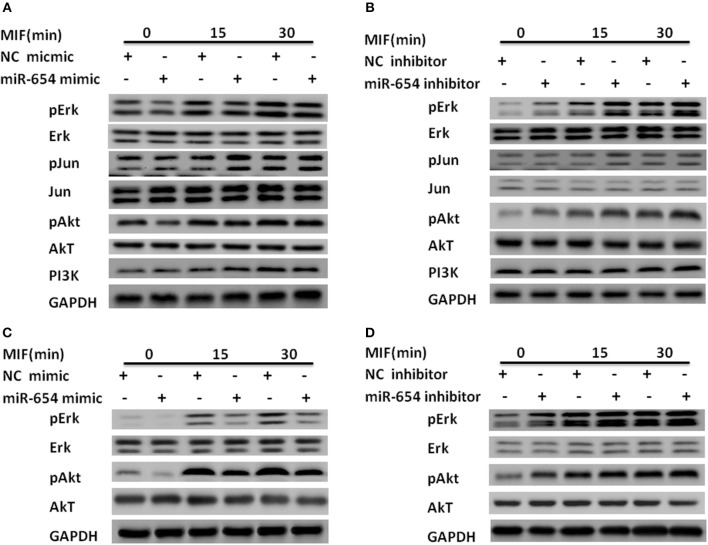
miR-654 inhibits inflammatory cytokine expression and cell proliferation by reducing tyrosine phosphorylation of ERK and AKT in RAW264.7 or THP-1 derived macrophages. **(A)** miR-654 mimic reduces the phosphorylation of ERK and AKT. RAW264.7 macrophages were transfected with miR-654 mimic or negative control (NC) mimic. After 24 h, cells were stimulated with MIF (100 ng/ml) for 15 or 30 min. **(B)** miR-654 inhibitor increased the phosphorylation of ERK and AKT. RAW264.7 were transfected with miR-654 inhibitor or negative control (NC) inhibitor, and stimulated after 24 h with MIF (100 ng/ml) for 15 or 30 min. **(C,D)** Macrophages differentiated from human THP-1 cells by PMA (100 ng/ml, 24 h) were transfected with miR-654 mimic, NC mimic, miR-654 inhibitor, or NC inhibitor. Cells were stimulated with MIF (100 ng/ml) for 15 or 30 min before harvesting for analysis.

Macrophage migration inhibitory factor (MIF) binds to its transmembrane receptor CD74 to activate CD44, or binds to other receptors, CXCR4 and CXCR2, and initiates a signaling cascade mediated by phosphorylation of ERK, Jun, and PI3K/AKT. These cytosolic elements induce expression of pro-inflammatory cytokines such as IL-1β, IL-6, IL-8, and TNFα. We examined whether miR-654 influences the expression of phosphorylation ERK, Jun, or PI3K/AKT, which may explain the molecular mechanism of miR-654. Tyrosine phosphorylation of ERK and AKT were significantly decreased in MIF stimulated RAW264.7 macrophages after overexpression of the miR-654 mimic, while total ERK, pJun, Jun, AKT, and PI3K protein levels were not significantly affected (Figures 4A,B; Supplementary Figure 2). As MIF and inflammatory cytokines play important roles in the pathogenesis of both mouse and human lupus nephritis, we also tested whether miR-654 activated the phosphorylation of ERK and AKT in human THP-1 derived macrophages. We found that miR-654 mimic decreased and miR-654 inhibitor increased the tyrosine phosphorylation of ERK and AKT (Figures 4C,D; Supplementary Figure 3). Protein levels of ERK, pJun, Jun, AKT, and PI3K were not significantly influenced. According to these results, we conclude that miR-654 down-regulates inflammatory cytokines expression by inhibiting MIF-dependent ERK and AKT phosphorylation.

Since exogenous MIF was not affected by miR-654, how miR-654 can affect exogenous MIF signal transduction? We examined the expression of MIF receptors by qPCR, and found that among the four classical MIF receptors: CD74, CD44, CXCR2, CXCR4. Only the expression of CD44 is significantly decreased by overexpression of miR-654 (Supplementary Figure 4). Further analysis that CD44 is a target gene of miR-654. The results suggest that miR-654 not only regulate the expression of MIF, but also regulate the expression of MIF receptor CD44.

### miR-654 Mimic Alleviates Lupus Nephritis in Pristine Induced Lupus Mice

**Figure 5:**
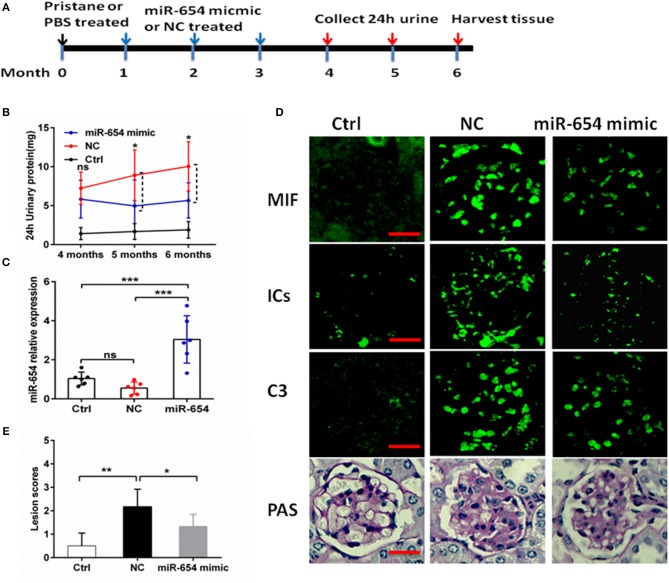
Efficacy of microRNA-654 (miR-654) overexpression in preventing pristine-induced lupus nephritis. **(A)** Design of animal experiments. **(B)** Twenty-four hour urinary protein expression levels were reduced after treated with miR-654 mimic in the fifth month after induced by pristine. **(C)** qPCR was used to detect miR-654 expression in renal tissues from control mice or from pristine-induced mice injected intravenously with miR-654 mimic or negative control (NC). **(D)** MIF, immune complexes (ICs) and C3 analyzed by immunofluorescence. Periodic acid–Schiff (PAS)–stained kidney sections were used to analyze renal lesion (*n* = 6), Bar = 25 μm. **(E)** Renal lesion scores, scored as described in Zhou et al. (17). **(B,C,E)** Were analyzed by Mann-Whitney test. *P < 0.05; **P < 0.01; ***P < 0.001 vs. negative control. ns, no significant.

As shown above, miR-654 can suppress the expression of MIF and inflammatory cytokines. In order to investigate the biological relevance of miR-654 *in vivo*, miR-654 mimic or NC were mixed with Lipofectamine® RNAiMAX and injected into lupus mice induced with pristine. Mice were sacrificed 6 months later to analyze renal pathology and serum autoantibodies, complement, and cytokines (Figure 5A). Pristine-induced lupus mice showed increased urinary protein, however the 24 h urinary protein is significantly decreased in the miR-654 group when compared with NC group 5 month after induced by pristine(Figure 5B, *P* < 0.05). miR-654 expression was significantly increased in the kidney in the miR-654 group(*P* < 0.05, Figure 5B), which indicated that miR-654 mimic had been successfully transfected into mice. Serum MIF expression was significantly lower in the miR-654 treated group (Figure 5C). Immunofluorescence analysis of renal sections obtained at the end of the study showed that miR-654 overexpression was associated with a decline in MIF expression in lupus glomeruli (Figure 5D). Moreover, the deposition of immune complexes and C3 in glomeruli was significantly lessened in the miR-654 treated mice when compared with NCs. miR-654 also reduced overall renal lesion scores when compared with the control (Figure 5E).

### miR-654 Inhibitor Aggravates Lupus Nephritis Induced by Pristine

**Figure 6:**
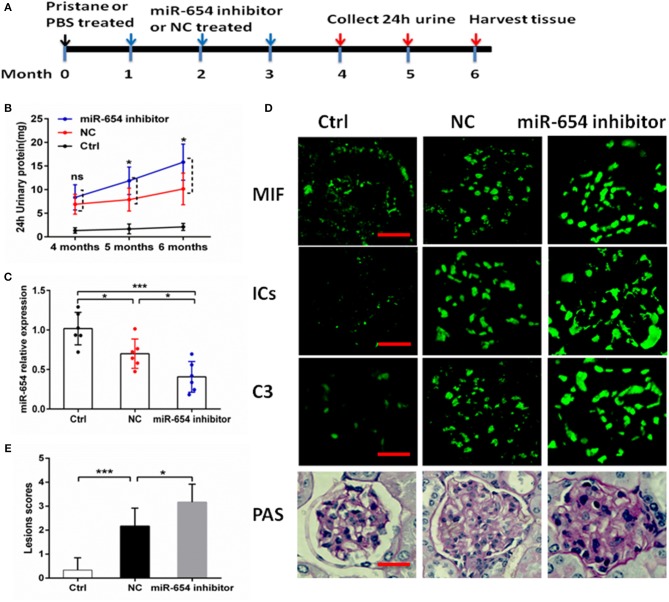
Efficacy of inhibit miR-654 expression in aggravating pristine-induced lupus nephritis. **(A)** Design of animal experiments. **(B)** Twenty-four hour urinary protein expression increased significantly in the fifth month after induced by pristine in miR-654 inhibitor group. **(C)** miR-654 expression reduced significantly in renal tissues from mice treated with miR-654 inhibitor compared with NC. **(D)** MIF, immune complexes (ICs) and C3 analyzed by immunofluorescence. Periodic acid–Schiff (PAS)–stained kidney sections were used to analyze renal lesion (*n* = 6), Bar = 25 μm. **(E)** Renal lesion scores obtained as described in Zhou et al. (17). **(B,C,E)** Were analyzed by Mann-Whitney test. *P < 0.05; ***P < 0.001 vs. negative control. ns, no significant.

**Figure 7:**
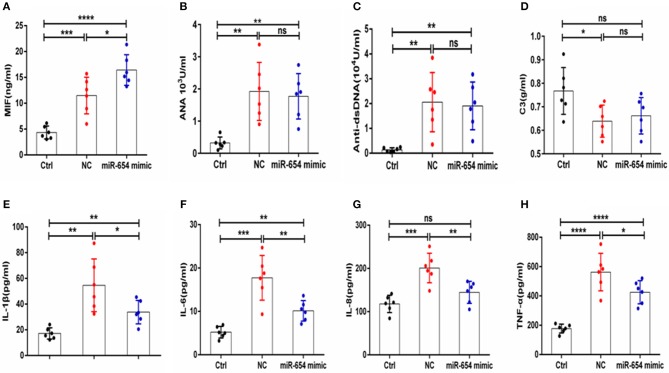
MiR-654 inhibited inflammatory cytokine expression in pristine-induced lupus mice model. **(A)** MIF expression levels was significantly reduced in miR-654 mimic treated mice. **(B–D)** ANA, anti-dsDNA, and C3 in the miR-654 and NC treated groups showed no significantly different. **(E–H)** The protein expression levels of IL-1β, IL-6, IL-8, and TNF-α in miR-654 vs. NC treated groups determined by ELISA. All data in this figure were analyzed by Mann-Whitney test. *P < 0.05; **P < 0.01; ***P < 0.001; ****P ≤ 0.0001 vs. negative control. ns, no significant.

In order to better understand the role of miR-654 in the pathogenesis of SLE, we treated mice with pristine 4 weeks before treating them with the miR-654 inhibitor (Figure 6A). Five months after induction by pristine, 24 h urinary protein increased significantly in miR-654 inhibitor group compared with NC group (*P* < 0.05, Figure 6B). miR-654 expression was significantly reduced in the kidney in miR-654 inhibitor group (*P* < 0.05, Figure 7B). Serum MIF expression was significantly increased in the miR-654 inhibitor treated group (Figure 6C). Immunofluorescence results showed that MIF expression also was increased in the renal tissues after inhibition of miR-654 expression (Figure 6D). Moreover, the deposition of immune complexes and C3 in glomeruli was significantly increased in the miR-654 inhibitor treated mice when compared with NC. miR-654 inhibitor also increased overall renal lesion scores when compared with controls (Figure 6E). These observations support the protective effect of miR-654 in pristine-induced lupus renal pathology and are consistent with prior reports of pharmacologic MIF antagonism in the distinct NZB/NZW F1 and MRL/*lpr* lupus models (13).

### miR-654 Plays an Important Role in the Pathogenesis of Lupus Nephritis by Regulation the Expression of Inflammation Cytokines

Sera MIF expression was reduced in miR-654 treated group mouse (*P* < 0.05, Figure 7A), and the expression of the inflammatory cytokines IL-1β, IL-6, IL-6, and TNF-α were also significantly reduced in the miR-654 treated group when compared with the NC group (Figures 7E–H). ANA, anti-dsDNA antibodies, and C3 were measured by ELISA and no significant differences were found between miR-654 group and NC group (Figures 7B–D).

**Figure 8:**
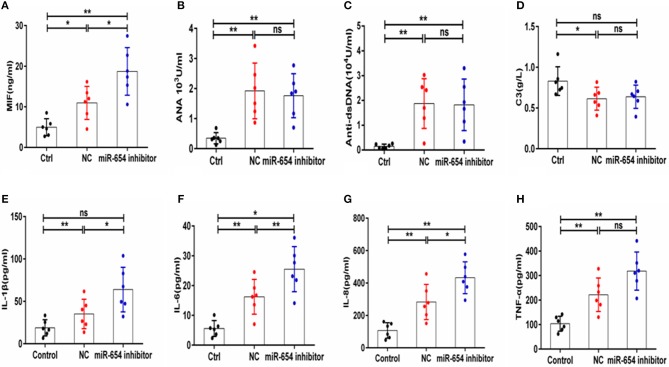
MiR-654 inhibitor promoted inflammatory cytokine expression in pristine-induced lupus mice model. **(A)** MIF expression levels was significantly increased in miR-654 inhibitor treated mice. **(B–D)** ANA, anti-dsDNA, and C3 in the miR-654 inhibitor and NC treated groups showed no significantly difference. **(E)** The protein expression levels of IL-1β, IL-6, IL-8, and TNF-α in miR-654 inhibitor vs. NC treated groups determined by ELISA. All data in this figure were analyzed by Mann-Whitney test. *P < 0.05; **P < 0.01 vs. negative control. ns, no significant.

The expression of serum MIF was significantly increased in the miR-654 inhibitor treated mouse when compared with the NC group (*P* < 0.05, Figure 8A), and the expression levels of IL-1β, IL-6, IL-6, and TNF-α were significantly increased in the miR-654 inhibitor treated group (Figures 8E–H). The expression of ANA, anti-dsDNA antibodies and C3 were significantly changed in the pristine-treated mice, while no significant differences were found between the miR-654 inhibitor group and the NC group (Figures 8B–D).

### Circulating MIF Protein Level Is Negatively Correlated With miR-654 Expression in SLE Patients

**Figure 9:**
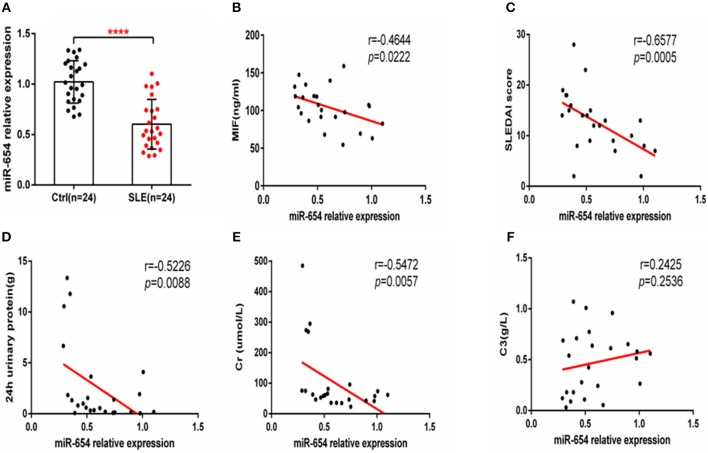
MIF and miR-654 expression are negatively correlated in SLE patients. miR-654 expression was decreased in PBMCs from SLE patients and correlates negatively with SLEDAI score. **(A)** Quantitative polymerase chain reaction (qPCR) analysis of miR-654 expression in PBMCs from SLE patients (*n* = 24) compared with healthy controls (*n* = 24). **(B)** miR-654 expression in PBMCs from SLE patients was negatively correlated with sera MIF expression(*n* = 24). **(C)** miR-654 expression levels from SLE patients correlates negatively with SLEDAI score. **(D)** miR-654 expression levels from SLE patients correlates negatively with 24 h urinary protein (*n* = 24). **(E)** miR-654 expression levels from SLE patients correlates negatively with sera creatine (*n* = 24). **(F)** miR-654 expression levels from SLE patients neither has negatively nor has positively relationship with C3 (*n* = 24). **(A)** was analyzed by Unpaired student's *t*-test. **(B–F)** were analyzed by nonparametric Spearman's correlation. ****P ≤ 0.0001 vs. negative control.

Given that miR-654 can regulate the expression of MIF and no study has reported the function or expression of miR-654 in SLE, we examined the expression of miR-654 in SLE patients and analyzed its relationship with clinical characteristics. MiR-654 expression was significantly reduced in SLE patients (*P* < 0.0001, Figure 9A) and a positive correlation between miR-654 expression and SLEDAI score in SLE patients was also observed (*P* < 0.001, Figure 9C). The circulating MIF protein level was negatively correlated with miR-654 expression (*P* < 0.01, Figure 9B). Moreover, we found that both 24 h urinary protein and serum creatinine negatively correlated with miR-654 expression (*P* < 0.05, Figures 9D,E). No significant positive or negative relationship was observed between C3 and MIF expression (Figure 9F).

## Discussion

Renal involvement is very common in SLE and severe lupus nephritis is a leading cause of morbidity and ultimate mortality. Autoantibody and immune complex deposition in the microvasculature of the kidney creates a proinflammatory microenvironment that leads to irreversible glomerular injury, tubular inflammation, and loss of function (21). MIF is an important upstream regulator of innate immunity that promotes the expression of pro-inflammatory cytokines such as TNF-α, IL-1β, IL-6, and IL-8 by suppressing activation-induced apoptosis (22) and counter-regulating the protective effects of endogenous or pharmacologic glucocorticoids (5, 7, 18, 24). Prior studies have demonstrated the importance of MIF in the pathogenesis of SLE and lupus nephritis (12, 13, 25). These data include the association between high expression *MIF* alleles and renal injury (7), the ameliorative effect of immunoneutralization or genetic deletion of MIF on experimental lupus nephritis (12, 13), and the overexpression of the MIF receptor CD74 in glomerulonephritis (26), all observations that have contributed to the scientific rationale for the ongoing clinical evaluation of a humanized anti-MIF receptor (anti-CD74) (27). Nevertheless, the precise pathogenic mechanisms for MIF's role in lupus nephritis remain to be clarified.

Morand et al. have reported that MIF expression is higher in Asian than other ethnic SLE cohorts (6). All SLE patients included in this report were Chinese, we found that both MIF mRNA expression level in PBMCs and MIF level in sera were significantly higher in SLE subjects than healthy controls, and verified that circulating MIF protein level positively correlated with SLEDAI score. Perhaps the Asian ethnic SLE cohorts may contributed to the high expression of MIF in our research.

Previous studies have demonstrated that miRNAs play important role in the pathogenesis of SLE. For example, miR-146a activates the type I IFN pathway in human lupus by targeting the key signal transducer Stat1 (16). miR-150 promotes renal fibrosis in lupus nephritis by downregulating suppressor of cytokine signaling 1 (SOCS1), which leads to increased expression of profibrotic molecules in renal tissue (17). Shen et al. have reported that miR-130b can ameliorate pristine-induced LN by interfering with the type I interferon pathway (18). We report a novel pathway whereby regulatory miRNAs (e.g., miR-451a, miR-654, and miR-152) inhibit MIF expression. Several reports have shown that miR-451a suppresses proliferation, migration and promotes the apoptosis of tumor cell by targeting MIF (21, 28–32). We report two new miRNAs, miR-654 and miR-152, that regulate the expression of MIF and have not been described previously to play a role in autoimmune rheumatic diseases. Previous work has implicated miR-654 in facioscapulohumeral muscular dystrophy (33) and Down syndrome (34). Huang et al. have reported that miR-654 can inhibit H1N1 influenza A virus replication by interaction with the PB1 gene (35). As in our study, miR-654 showed the most powerful ability to inhibit the expression of MIF among candidate miRNAs, we focused on its potential role in pro-inflammatory pathways relevant to lupus. Our data showed that miR-654 was significantly downregulated in the PBMCs of SLE patients and negatively correlated with SLEDAI scores. Furthermore, miR-654 expression negatively correlated with serum MIF level. We overexpressed miR-654 in RAW264.7 macrophages and found miR-654 to inhibit both MIF and the MIF-dependent downstream cytokines, such as IL-1β, IL-6, IL-8, and TNF-α. Furthermore, we found these effects associated with MIF dependent phosphorylation of ERK and AKT in both human and murine macrophages.

In the pristine-induced mouse model of SLE, the experimental administration of miR-654 did not influence circulating ANA and anti-dsDNA antibody levels, suggesting little impact on dysregulation of the adaptive response. Taken together, these results indicate that miR-654 may play a pivotal role in the progression of lupus nephritis by regulating pathologic MIF overexpression. These data also imply that miR-654 may have potential therapeutic application in lupus nephritis, particularly in individuals who are with high genotypic *MIF* expressers (7). Important questions to pursue further include a better definition of the potential therapeutic targets for miR-654 action, which could include not only specific immune cell subsets but renal stromal cells. In the case of the later, the targeted delivery of miR-654 into renal tissue may be feasible (36).

## Data Availability

The raw data supporting the conclusions of this manuscript will be made available by the authors, without undue reservation, to any qualified researcher.

## Ethics Statement

All animal experiments complied with the relevant institutional guidelines of animal ethics from Renji Hospital, Shanghai Jiao Tong University, School of Medicine.

## Author Contributions

All authors listed have made a substantial, direct and intellectual contribution to the work, and approved it for publication.

### Conflict of Interest Statement

The authors declare that the research was conducted in the absence of any commercial or financial relationships that could be construed as a potential conflict of interest.
