# Supplementary material for: MiRNA Regulation of MIF in SLE and Attenuation of Murine Lupus Nephritis With miR-654
Source: Front Immunol. 2019 Sep 19;10:2229. doi: 10.3389/fimmu.2019.02229 (PMC6761280; doi:10.3389/fimmu.2019.02229)
Supplement: Supplementary file 1 [file Data_Sheet_1.PDF]

Supplementary Table 1: The sequence of the mimic, inhibitor, and control

| Name | Direction | Sequence |
|---|---|---|
| miR-148b | sense(5'-3') | UCAGUGCAUCACAGAACLLLGU |
| | antisense(5'-3') | AAAGUUCUGUGAUGCACUGAUU |
| miR-451a | sense(5'-3') | AAACCGUUACCAUUACUGAGUU |
| | antisense(5'-3') | CUCAGUAAUGGUAACGGUUUUU |
| miR-654 | sense(5'-3') | UGGUGGGCCGCAGAACAUGUGC |
| | antisense(5'-3') | ACAUGUUCUGCGGCCCACCAUU |
| miR-608 | sense(5'-3') | AGGGGUGGUGUUGGGACAGCUCCGU |
| | antisense(5'-3') | GGAGCUGUCCCAACACCACCCCUUU |
| miR-629 | sense(5'-3') | GUUCUCCCAACGUAAGCCCAGC |
| | antisense(5'-3') | UGGGCUUACGUUGGGAGAACUU |
| miR-363-5p | sense(5'-3') | CGGGUGGAUCACGAUGCAAUUU |
| | antisense(5'-3') | AUUGCAUCGUGAUCCACCCGUU |
| miR-152 | sense(5'-3') | UCAGUGCAUGACAGAACUUGGG |
| | antisense(5'-3') | CAAGUUCUGUCAUGCACUGAUU |
| miR-31-5p | sense(5'-3') | AGGCAAGAUGCUGGCAUAGCU |
| | antisense(5'-3') | CUAUGCCAGCAUCUUGCCUUU |
| miR-504-5p | sense(5'-3') | AGACCCUGGUCUGCACUCUAUC |
| | antisense(5'-3') | UAGAGUGCAGACCAGGGUCUUU |
| miR-92a-1-5p | sense(5'-3') | AGGUUGGGAUCGGUUGCAAUGCU |
| | antisense(5'-3') | CAUUGCAACCGAUCCCAACCUUU |
| miR-1247-3p | sense(5'-3') | CCCCGGGAACGUCGAGACUGGAGC |
| | antisense(5'-3') | UCCAGUCUCGACGUUCCCGGGGUU |
| miR-6819-5p | sense(5'-3') | UUGGGGUGGAGGGCCAAGGAGC |
| | antisense(5'-3') | UCCUUGGCCCUCCACCCCAAUU |
| miR NC | sense(5'-3') | UUCUCCGAACGUGUCACGUTT |
| | antisense(5'-3') | ACGUGACACGUUCGGAGAATT |
| miR-654 inhibitor | sense(5'-3') | ACACAUGUUCUGCGGCCCACCA |
| miR inhibitor NC | sense(5'-3') | CAGUACUUUUGUGUAGUACAA |
