# Supplementary material for: MiRNA Regulation of MIF in SLE and Attenuation of Murine Lupus Nephritis With miR-654
Source: Front Immunol. 2019 Sep 19;10:2229. doi: 10.3389/fimmu.2019.02229 (PMC6761280; doi:10.3389/fimmu.2019.02229)
Supplement: Supplementary file 2 [file Data_Sheet_2.PDF]

Supplementary Table 2: The binding sites between miRNAs and MIF

<table>
<tr><th></th><th>Predicted consequential pairing of target region (top) and miRNA (bottom)</th><th>Site type</th></tr>
<tr><td>Position 71-77 of MIF 3' UTR
hsa-miR-654-5p</td><td>5' ...CUGUGUUCUAGGCCCGCCCACCC...
|||||| |||||||
3' CGUGUACAAGACGC——CGGGUGGU</td><td>7mer-m8</td></tr>
<tr><td>Position 92-99 of MIF 3' UTR
hsa-miR-629-3p</td><td>5' ...CCCAACCUUCUGGUGGGAGAAA...
||| |||||||
3' CGACCCGAAUGCAA-CCCUCUUG</td><td>8mer</td></tr>
<tr><td>Position 102-108 of MIF 3' UTR
hsa-miR-451a</td><td>5' ...UGGUGGGGAGAAAUAAACGGUUU...
|||||||
3' UUGAGUCAUUACCAUUGCCAAA</td><td>7mer-m8</td></tr>
<tr><td>Position 77-83 of MIF 3' UTR
hsa-miR-92a-1-5p</td><td>5' ...UCUAGGCCCGCCCACCCCAACCU...
|||||||
3' UCGUAACGUUGGCUAGGGUUGGA</td><td>7mer-m8</td></tr>
<tr><td>Position 73-80 of MIF 3' UTR
hsa-miR-608</td><td>5' ...GUGUUCUAGGCCCGC—CCACCCCA...
||| |||||||
3' UGCCUCGACAGGGUUGUGGUGGGGA</td><td>8mer</td></tr>
<tr><td>Position 37-43 of MIF 3' UTR
hsa-miR-1247-3p</td><td>5' ...UCUGCGCUGGCUCCACCCGGGAA...
||||||
3' CGAGGUCAGAGCUGCAAGGGCCCC</td><td>7mer-A1</td></tr>
<tr><td>Position 74-81 of MIF 3' UTR
hsa-miR-6819-5p</td><td>5' ...UGUUCUAGGCCCGCCCACCCCAA...
||||| |||||||
3' CGAGGAACCGGGAG-GUGGGGUU</td><td>8mer</td></tr>
<tr><td>Position 33-39 of MIF 3' UTR
hsa-miR-363-5p</td><td>5' ...GCUGUCUGCGCUGGCUCCACCCG...
|||||||
3' UUUAACGUAGCACUAGGUGGGC</td><td>7mer-m8</td></tr>
</table>

Position 79-88 of MIF 3'UTR 5' CCCCAACCTTCTGGTGGGG
||| |||||
has-miR-148-3p 3' TGTTTCAAGACACTACGTGACT

Position 19-30 of MIF 3'UTR 5' CGCTGTC TGCGCTGGCTC
||||| ||| |||
has-miR-148-3p 3' GGTTCAAGACAGTACGTGACT
