# Supplementary figures and images for: MiRNA Regulation of MIF in SLE and Attenuation of Murine Lupus Nephritis With miR-654
Source: Front Immunol. 2019 Sep 19;10:2229. doi: 10.3389/fimmu.2019.02229 (PMC6761280; doi:10.3389/fimmu.2019.02229)

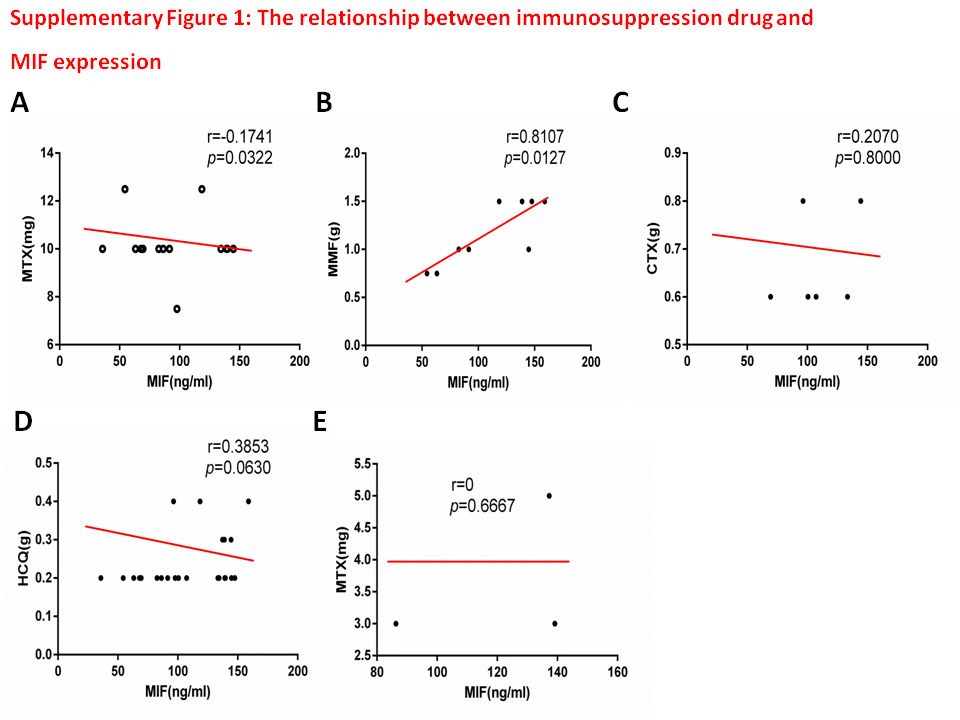

Supplement: Supplementary file 3 [file Image_1.TIF]

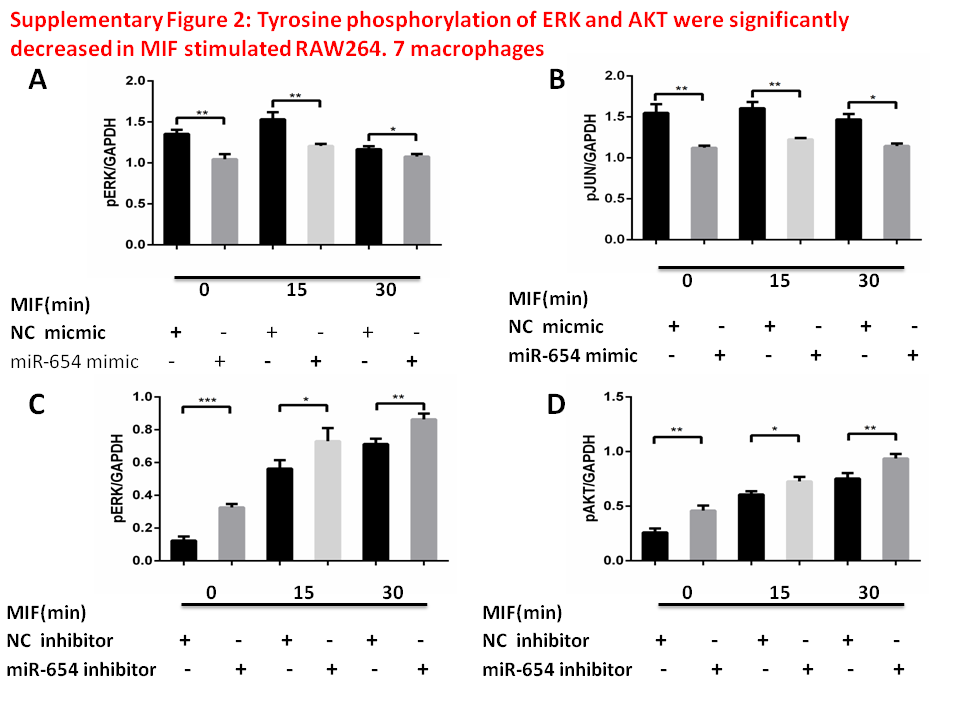

Supplement: Supplementary file 4 [file Image_2.TIF]

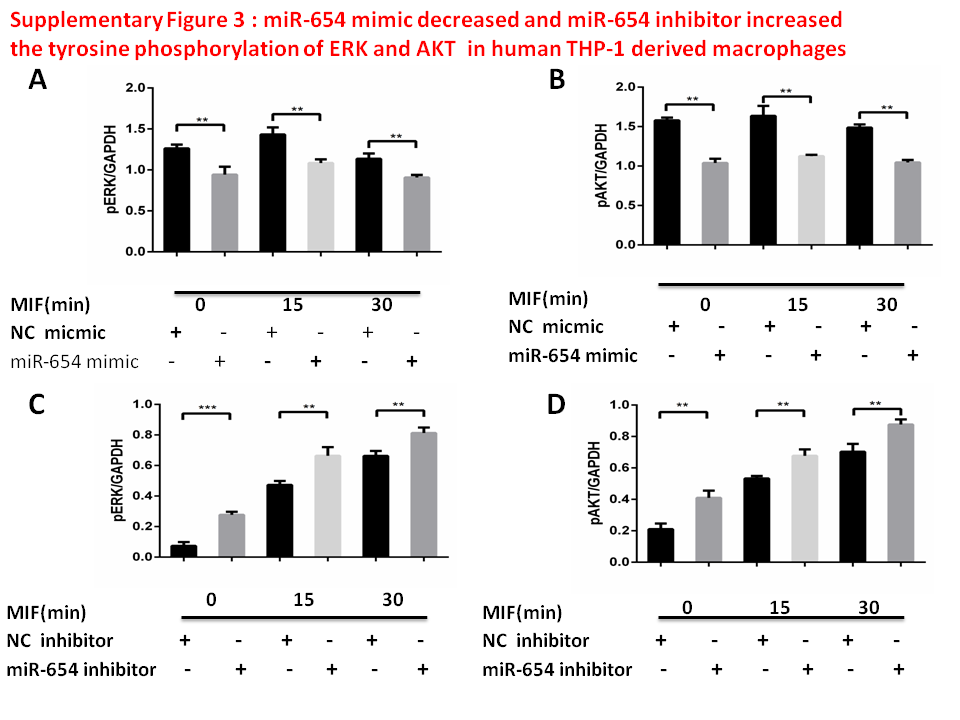

Supplement: Supplementary file 5 [file Image_3.TIF]

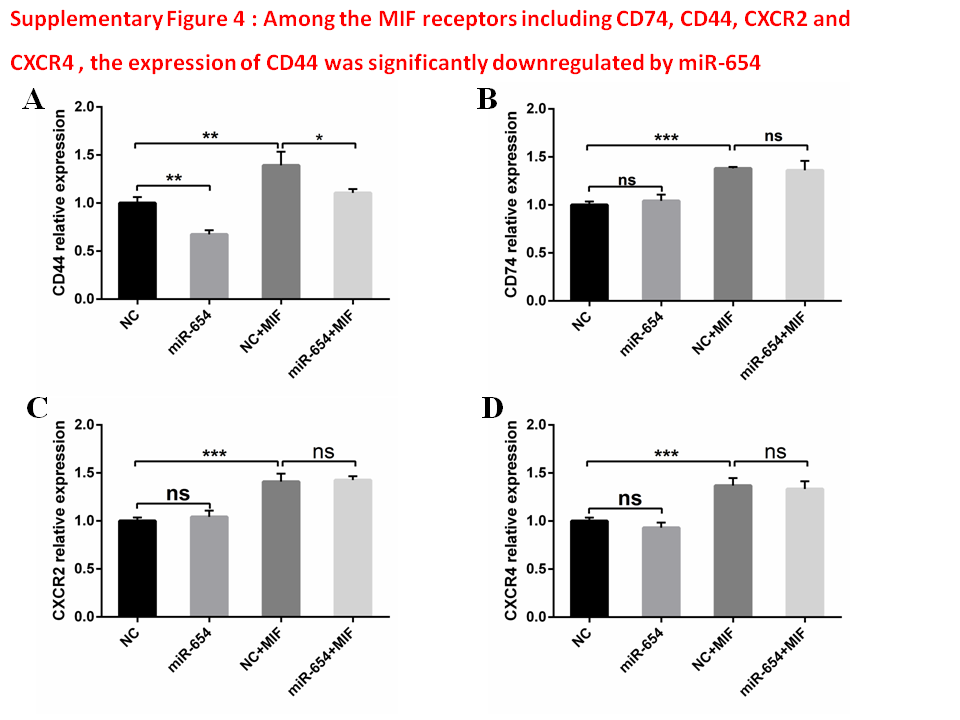

Supplement: Supplementary file 6 [file Image_4.TIF]
